# Macrolide-resistant *Mycoplasma pneumoniae* in children: clinical features, treatment response, and outcomes in a retrospective cohort from China

**DOI:** 10.3389/fped.2026.1812564

**Published:** 2026-04-20

**Authors:** Lulu Zheng, Huaqing Liu, Cunxin Xu, Feifei Song, Shenggang Ding

**Affiliations:** 1 Department of Pediatrics, Anhui Zhongke Gengjiu Hospital, Hefei, Anhui Province, China; 2 Department of Pediatrics, First Affiliated Hospital of Anhui Medical University, Hefei, China; 3 Anhui Provincial Children’s Hospital, Hefei, Anhui Province, China

**Keywords:** 23S rRNA mutations, antimicrobial resistance, clinical outcomes, minimal inhibitory concentration, *Mycoplasma pneumoniae*, pediatric pneumonia

## Abstract

**Introduction:**

Macrolide-resistant *Mycoplasma pneumoniae* (MRMP) has reached extremely high prevalence among children in Asia. However, genotype -phenotype correlations and their impact on clinical outcomes in pediatric community-acquired pneumonia (CAP) remain insufficiently characterized in many regions of China. This study aimed to determine the prevalence, molecular mechanisms, antimicrobial susceptibility, and clinical characteristics of macrolide resistance in children with M. *pneumoniae* CAP in Anhui, China.

**Methods:**

A retrospective cohort study was conducted among 71 pediatric patients with confirmed M. *pneumoniae* CAP between July 2023 and July 2024. Macrolide resistance was assessed using 23S rRNA domain V sequencing and broth microdilution minimum inhibitory concentration (MIC) testing. Clinical features, laboratory markers, treatment response, and outcomes were descriptively compared between macrolide-resistant M. pneumoniae (MRMP) and macrolide-susceptible M. *pneumoniae* (MSMP) cases.

**Results:**

Of the 71 isolates, 67 (94.4%) were macrolide-resistant, predominantly harboring the A2063G mutation (81.7%). The MIC₅₀/MIC₉₀ values for erythromycin and azithromycin were 126/512 μg/mL and 16/126 μg/mL, respectively. Compared with the small MSMP group (*n* = 4), children with MRMP appeared to have longer median fever duration (6.5 vs. 4.0 days), longer hospitalization (7.0 vs. 5.0 days), higher hs-CRP (11.2 vs. 4.8 mg/L), higher LDH (258.5 vs. 210.3 U/L), more persistent cough, delayed radiographic resolution, and higher treatment-failure or antibiotic-switch rates (20.9% vs. 0%).

**Discussion:**

Macrolide resistance exceeded 94% and was mainly driven by the A2063G mutation, accompanied by high MIC values. MRMP infections were associated with prolonged clinical course and elevated inflammatory markers; however, these findings should be interpreted cautiously due to the extremely small comparator group and the descriptive nature of the analysis.

## 1 Introduction

Community-acquired pneumonia (CAP) is one of the most common infectious diseases worldwide and continues to represent a major cause of morbidity and mortality in both children and adults (1). Among the causative agents, *Mycoplasma pneumoniae* (MP) is an atypical bacterial pathogen that has been recognized as an important cause of respiratory tract infections, including up to 10%–40% of CAP cases in different countries (2). In China, surveillance studies suggest that MP accounts for a considerable proportion of both pediatric and adult CAP cases, with seasonal and epidemic fluctuations observed in recent years (3). Because MP lacks a cell wall, it is resistant to *β*-lactams and aminoglycosides, making macrolides the standard first-line therapy in most clinical settings (4).

Macrolides such as erythromycin and azithromycin are widely used due to their antimicrobial and immunomodulatory effects and are especially favored in children, where tetracyclines and fluoroquinolones are not routinely recommended because of safety concerns (5–7). However, the emergence of macrolide-resistant *MP* (MRMP) has become a growing public health problem over the past two decades (8). The prevalence of MRMP has risen dramatically in Asia, particularly in China and Japan, where resistance rates as high as 80%–90% have been reported in some regions. Resistance rates in Europe and North America are generally lower but are also increasing, Underscoring the global nature of the problem (9).

Children represent the majority of community-acquired *MP* cases, and macrolides are often the only safe therapeutic option because tetracyclines and fluoroquinolones are contraindicated in this age group (10). As a result, when resistance emerges, treatment failures are more likely, leading to prolonged symptoms, increased hospitalizations, and greater healthcare burden (11). Monitoring resistance patterns in pediatric cases is therefore critical for guiding therapy and minimizing adverse outcomes (12).

The primary mechanism of macrolide resistance in MP involves point mutations in domain V of the 23S ribosomal RNA (rRNA) gene, most commonly the A2063G and A2064G transitions (13). These mutations reduce the binding affinity of macrolides to the ribosomal subunit, leading to high-level resistance. Additional mutations, such as A2067G, A2063C, and C2617G, have also been described and are associated with varying levels of resistance (14, 15). Importantly, MRMP infections have been associated with prolonged fever, persistent cough, delayed radiographic resolution, and longer hospital stays compared to infections with susceptible strains (16). This highlights the clinical relevance of resistance beyond *in vitro* findings.

Resistance to macrolides has become increasingly prevalent in China, with many isolates exhibiting high MIC values (17, 18). Nevertheless, β-lactams such as ceftriaxone are still often prescribed empirically in pediatric pneumonia, despite their lack of activity against MP (19). These limitations underscore the restricted therapeutic options available for children and highlight the importance of monitoring resistance trends and linking microbiological findings with clinical outcomes.

Epidemiological surveillance of antimicrobial resistance in MP is therefore essential to guide clinical decision-making, inform empirical treatment strategies, and prevent the unchecked spread of resistant strains (20). Although several studies have been conducted in China, most data are derived from adult populations or limited geographic areas. Fewer large-scale studies have addressed MRMP prevalence and clinical impact in pediatric patients or across diverse regions. Moreover, while resistance patterns have been described, relatively fewer studies integrate both clinical characteristics, laboratory, and molecular determinants, limiting our understanding of how genotypic findings correlate with clinical outcomes. This gap is particularly important in China, where high antibiotic consumption and variable prescribing practices may contribute to regional differences in resistance prevalence (21). Understanding local patterns of antimicrobial susceptibility and genetic mutations in MP isolates is critical for tailoring treatment recommendations and anticipating future resistance trends (22). In addition, examining the clinical features of patients with MRMP and those with macrolide-susceptible MP can help clinicians recognize resistant infections earlier and adjust therapy accordingly.

The present study was designed to investigate clinical features, laboratory markers, treatment responses, and resistance patterns in pediatric MP infections in China using a retrospective cohort. While several studies in China have reported high prevalence of MRMP, most have focused primarily on resistance rates or molecular characterization. Fewer studies have systematically integrated antimicrobial susceptibility profiles, resistance-associated mutations, and detailed clinical outcomes in pediatric cohorts. This study addresses this gap by linking genotype, phenotype, and clinical response within a single cohort, thereby providing a more comprehensive assessment of the clinical implications of macrolide resistance.

## 2 Materials and methods

### 2.1 Study design and population

**Figure 1:**
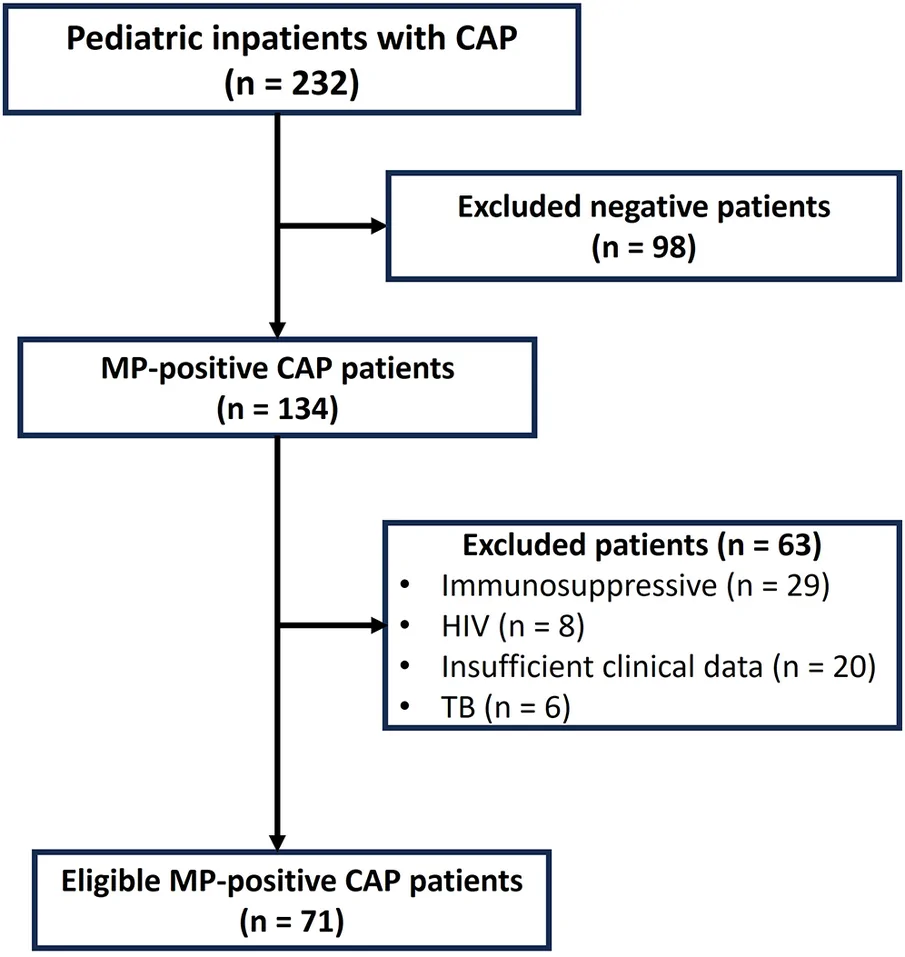
Flow diagram of pediatric CAP patients enrolled with *Mycoplasma pneumoniae*.

This retrospective single-center clinical study was conducted among pediatric patients diagnosed with CAP who attended Anhui Zhongke Gengjiu Hospital in Anhui, China, between July 2023 and July 2024. Eligible participants were children with clinical symptoms and chest radiographic findings consistent with CAP and a confirmed diagnosis of MP infection by culture and/or polymerase chain reaction (PCR). Patients were consecutively enrolled among hospitalized pediatric CAP cases during the study period. Exclusion criteria included insufficient clinical data, immunocompromised status, including HIV infection, ongoing immunosuppressive therapy or known/suspected active pulmonary tuberculosis. Mild or outpatient cases were not included in this cohort (Figure 1). This study included only hospitalized pediatric CAP patients with confirmed *M. pneumoniae* infection identified from hospital records during the study period. The dataset used for this analysis comprised only MP-positive cases, and information on the total number of CAP cases screened or the number of MP-negative cases was not available due to the retrospective nature of data collection.

### 2.2 Data collection

Demographic, clinical, and treatment information were extracted from medical records using a standardized data collection form. Variables included sex, age, hospitalization status, presenting symptoms such as cough, fever, breathlessness, chest pain, hemoptysis, comorbidities, initial laboratory findings, and antibiotic therapy initiated within 72 h of presentation. Disease severity was assessed using the CURB-65 score. Treatment failure was defined according to the Chinese guideline on treating (23) as persistence or worsening of fever or respiratory symptoms ≥72 h after initiating macrolide therapy, necessitating modification or addition of alternative antibiotics. This definition may be nonspecific in *M. pneumoniae* infection, as persistent symptoms can also reflect host immune response, disease progression, or co-infection independent of antimicrobial resistance. Adjunctive antibiotics were prescribed in cases of suspected bacterial co-infection or prolonged fever refractory to macrolide therapy. However, we acknowledge that persistent symptoms may also reflect host immune response, co-infection, or disease progression independent of antimicrobial resistance.

### 2.3 Microbiological testing of MP

At enrollment, throat swab specimens were collected from all patients for parallel culture and PCR testing to detect *M. pneumoniae*. Following collection, each swab was placed into 2 mL of transport medium and vortexed; then a 200 µL aliquot was inoculated into 1.8 mL of liquid *Mycoplasma* growth medium and incubated at 37 °C. Growth was monitored by the acidification of glucose in the medium: when viable MP ferment glucose, the phenol red indicator shifts from red to yellow. Cultures showing color change within 1–6 weeks were considered positive for MP.

### 2.4 Molecular analysis of resistance genes

DNA was extracted from throat swab specimens using a commercial kit (QIAamp DNA Mini Kit, Qiagen, Hilden, Germany) following the manufacturer’s protocol. The presence of *M. pneumoniae* DNA was first confirmed by real-time PCR using a CFX96™ Real-Time PCR Detection System (Bio-Rad, Hercules, CA, USA) (24). For resistance screening, probe-based PCR assays were used to rapidly detect the two most common 23S rRNA domain V mutations (A2063G and A2064G). To comprehensively characterize resistance, the entire domain V region of the 23S rRNA gene was subsequently amplified and sequenced, as previously described by Jiang et al. (25). Sequence data were aligned with the reference strain M129 (*M. pneumoniae* ATCC 29342) to identify known resistance-associated point mutations, including A2063G, A2064G, A2067G, and C2617G.

### 2.5 Antimicrobial susceptibility testing

*MP* isolates were propagated in specialized broth medium, and the minimal inhibitory concentrations (MICs) of selected antimicrobial agents were determined using a standard broth microdilution assay in accordance with Clinical and Laboratory Standards Institute (CLSI) recommendations (25). The antibiotics evaluated in this study were erythromycin (ERY), azithromycin (AZM), ceftriaxone (CRO), and amoxicillin–clavulanate (AMC). Breakpoints for macrolides were interpreted as follows: ERY, susceptible (S) ≤ 0.5 μg/mL, resistant (R) ≥ 1 μg/mL; AZM, S ≤ 0.5 μg/mL, R ≥ 1 μg/mL. For β-lactam antibiotics (CRO and AMC), interpretive criteria are not applicable since *MP* lacks a cell wall and is intrinsically resistant to this drug class; these agents were tested for completeness only, as they were frequently prescribed in the clinical cohort. MIC values were read as the lowest antibiotic concentration that inhibited visible color change in the culture medium. Reference strain M129 was included for quality control.

### 2.6 Statistical analysis

Statistical analysis was performed using SPSS IBM statistics 25.0 (IBM Corp., Armonk, NY, USA). Comparisons between MRMP and MSMP groups were conducted using non-parametric tests (Mann–Whitney U for continuous variables, Fisher’s exact test for categorical variables) due to the small comparator group. Effect sizes with 95% confidence intervals (CI) were calculated (median difference for continuous outcomes, risk difference for categorical outcomes). A two-sided *P* value <0.05 was considered statistically significant, but estimates were interpreted with caution given the limited sample size. No formal hypothesis testing framework was applied. Given the extremely small macrolide-susceptible group (MSMP, *n* = 4), all comparisons are presented as descriptive and exploratory. *P*-values are reported for completeness but do not support inferential conclusions.

## 3 Results

### 3.1 Patient characteristics

A total of 71 pediatric patients with confirmed MP infection were included in this study. The mean age of the cohort could not be calculated due to inconsistencies in age reporting, but most patients were between 3 and 12 years of age. Overall, 31 (43.7%) were male and 40 (56.3%) were female (Table 1). The median length of hospital stay was 6.5 days (interquartile range [IQR]: 6–8), with a range of 3–13 days. The most common discharge diagnosis was MP infection, recorded in 54 (76.1%) patients, followed by gastrointestinal dysfunction in 40 (56.3%), pneumonia in 27 (38.0%), and lobar pneumonia in 17 (23.9%). Several patients presented with overlapping diagnoses, reflecting the multisystem involvement often observed in pediatric cases.

**Table 1:** Baseline characteristics of pediatric patients with *Mycoplasma pneumoniae* infection.

Characteristics	Value
Total number of patients	71
Sex, *n* (%)	Male: 31 (43.7%), Female: 40 (56.3%)
Age, mean ± SD (range)	Mostly 3–12 years
Length of hospital stay, median (IQR)	6.5 days (6–8)
Length of hospital stay, range	3–13 days
Most common discharge diagnoses, n (%)	Mycoplasma infection: 54 (76.1%)
	Gastrointestinal dysfunction: 40 (56.3%)
	Pneumonia: 27 (38.0%)
	Lobar pneumonia: 17 (23.9%)

### 3.2 Clinical manifestations

At admission, the most frequent presenting symptoms included fever and cough, both documented in most patients. Several children exhibited signs of respiratory distress, particularly those with lobar pneumonia, and gastrointestinal complaints were also noted in a subset (Table 2). Radiological findings frequently demonstrated lobar or patchy pneumonia. At discharge, most patients showed marked clinical improvement, with resolution of fever and normalization of vital signs. A few children continued to experience mild residual cough, and delayed radiographic resolution was observed in a small proportion.

**Table 2:** Clinical manifestations of pediatric patients at admission and discharge.

Clinical features	Admitted	Discharged
Fever	Present in majority (>60% of cases)	Resolved in almost all patients
Cough	Common presenting symptoms (>70%)	Mild residual cough in some
Respiratory distress	Reported in subset with lobar pneumonia	Improved/disappeared
Chest imaging	Lobar or patchy pneumonia in several cases	Radiographic resolution delayed in a few
Other symptoms	Gastrointestinal complaints in some patients	Mostly resolved

### 3.3 Antibiotic prescription patterns

**Figure 2:**
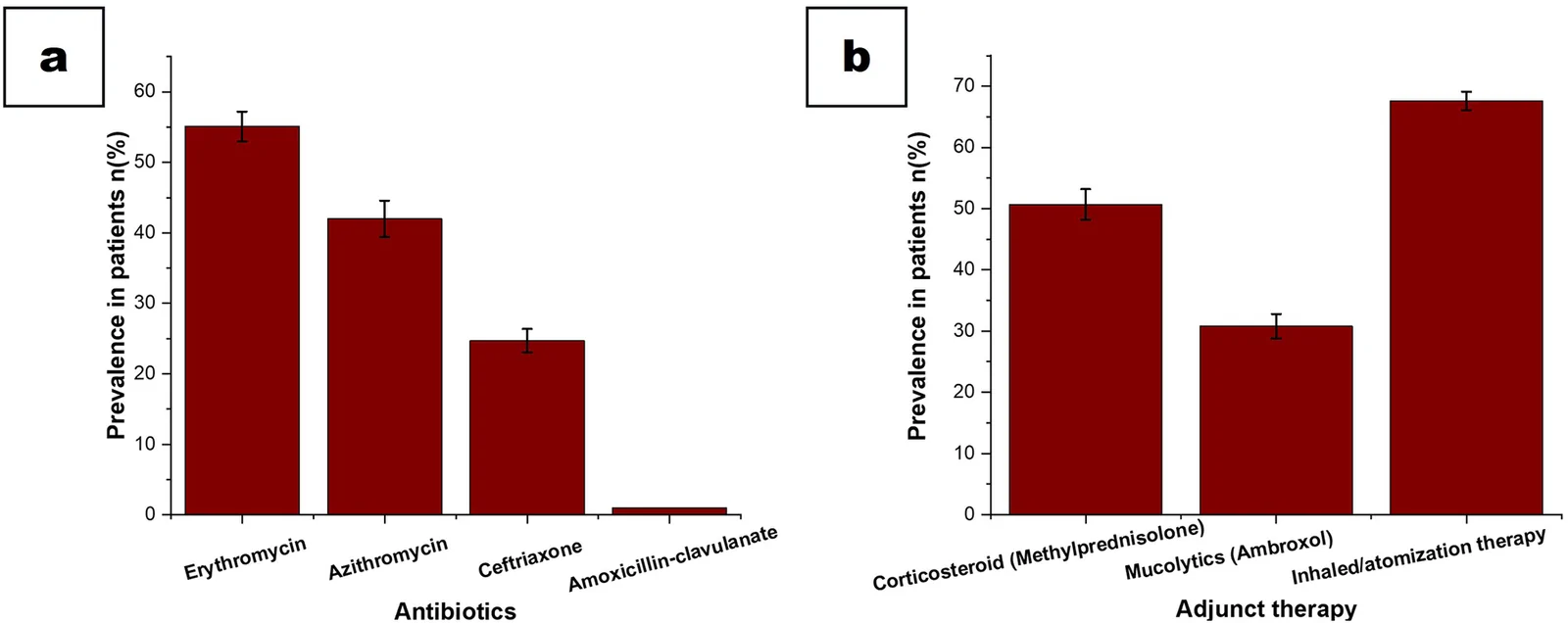
Antibiotic prescription patterns **(a)** and adjunctive therapies **(b)** in patients with *Mycoplasma pneumoniae* infection, expressed as prevalence (*n* = 71).

All patients received macrolides as first-line therapy. Erythromycin (10 mg/kg IV twice daily for approximately 6 days) was prescribed in 37 cases (52.1%), while azithromycin (10 mg/kg IV once daily or orally for 5–8 days) was used in 27 cases (38.0%). In addition, 42 patients (59.2%) received adjunctive antibiotics, most frequently ceftriaxone (40–80 mg/kg/day IV; 17 patients, 23.9%) or amoxicillin–clavulanate (30 mg/kg every 8 h; 1 patient, 1.4%) (Figure 2A).

Adjunctive therapies were also common. Corticosteroid treatment with methylprednisolone (1 mg/kg IV daily) was administered in 36 patients (50.7%), primarily in cases with more severe or persistent symptoms (Figure 2B). Supportive therapy included mucolytics such as ambroxol (29 patients, 40.8%) and inhaled agents including budesonide, terbutaline, or ipratropium (48 patients, 67.6%). Standard symptomatic management with antipyretics and intravenous fluids was provided to nearly all patients.

### 3.4 Antibiotic susceptibility

MIC distributions for the antibiotics administered in our cohort are shown in Table 3. Erythromycin exhibited a wide MIC range (0.016–512 μg/mL), with MIC50 and MIC90 values of 126 and 512 μg/mL, respectively. Azithromycin showed somewhat lower MICs (MIC50 16 μg/mL; MIC90 126 μg/mL), but resistance remained widespread. As expected for a cell wall–deficient organism, ceftriaxone and amoxicillin–clavulanate demonstrated uniformly high MICs (>256 μg/mL) and are reported only to contextualize empiric co-prescription in this cohort.

**Table 3:** MIC values of *M. pneumoniae* isolates for antibiotics recorded in cohort treatments.

Antibiotic	MIC range (*μ*g/mL)	MIC50 (μg/mL)	MIC90 (μg/mL)	MIC value of reference strain M129 (μg/mL)
ERY	0.016–512	126	512	0.004
AZM	0.008–256	16	126	0.004
CRO	64 – > 512	>256	>512	>256
AMC	128 – > 512	>256	>512	>256

ERY, erythromycin; AZM, azithromycin; CRO, ceftriaxone; AMC, amoxicillin–clavulanate. *β*-lactam MICs are presented for clinical context; *M. pneumoniae* lacks a cell wall and is intrinsically non-susceptible to *β*-lactams.

### 3.5 Genetic determinants of MP

The domain V region of the 23S rRNA gene identified macrolide resistance-associated mutations in the majority of isolates. Among 71 *M. pneumoniae* strains, 67 (94.4%) carried a resistance mutation, while 4 (5.6%) retained a wild-type sequence (Table 4). The A2063G transition was the predominant mutation, detected in 58 isolates (81.7%), followed by A2064G in 6 (8.5%), A2067G in 2 (2.8%), and C2617G in 1 (1.4%).

**Table 4:** Frequency of 23S rRNA mutations in pediatric *M. pneumoniae* isolates.

Mutation	n (%)	Clinical significance
A2063G	58 (81.7%)	High-level macrolide resistance
A2064G	6 (8.5%)	High-level resistance
A2067G	2 (2.8%)	Moderate resistance
C2617G	1 (1.4%)	Low-level resistance
Wild-type	4 (5.6%)	Macrolide-susceptible

### 3.6 Laboratory markers

Due to the limited size of the MSMP group, comparisons between MRMP and MSMP were interpreted as descriptive trends rather than definitive inferential findings. The overall blood routine and biochemical findings of the study cohort are presented in Table 5. Inflammatory markers were consistently elevated, with a median hs-CRP of 10.23 mg/L (IQR 5.07–15.60) and LDH of 244.9 U/L (IQR 224.5–290.5), reflecting a systemic inflammatory response and possible lung tissue injury. Hematological parameters showed modest alterations: lymphocyte percentages were slightly reduced (median 26.6%, IQR 21.6–33.9), eosinophil counts were suppressed, and platelet counts remained within the high-normal range (median 249.5 × 10^9/L). In contrast, liver function indices, including ALT, AST, and total bilirubin, as well as albumin and total protein levels, were largely within normal ranges.

**Table 5:** Baseline laboratory markers in pediatric patients with *Mycoplasma pneumoniae* infection.

Parameter	n	Median (IQR)	Mean ± SD	Reference trend
Eosinophils (%)	45	0.90 (0.30–2.20)	1.57 ± 1.84	Mostly low
Eosinophils (absolute, ×10^9/L)	45	0.07 (0.02–0.14)	0.11 ± 0.14	Suppressed
Lymphocytes (%)	45	26.6 (21.6–33.9)	27.9 ± 10.2	Slightly reduced
Platelets (×10^9/L)	46	249.5 (216.5–305.8)	268.4 ± 106.1	Within normal
hs-CRP (mg/L)	46	10.23 (5.07–15.60)	12.1 ± 11.2	Elevated in many
Procalcitonin (ng/mL)	1	0.08	–	Rarely tested
Total protein (g/L)	47	68.4 (65.9–69.9)	67.7 ± 6.6	Normal
Albumin (g/L)	47	41.7 (40.3–43.3)	41.9 ± 2.0	Normal
Total bilirubin (μmol/L)	47	6.1 (5.4–8.0)	6.7 ± 1.8	Normal
ALT (U/L)	47	12.9 (11.1–15.3)	17.4 ± 16.6	Mostly normal
AST (U/L)	47	31.1 (26.1–33.3)	32.4 ± 10.8	Normal–mild ↑
LDH (U/L)	47	244.9 (224.5–290.5)	256.9 ± 43.7	Mildly elevated
ALP (U/L)	47	181.8 (167.1–207.5)	206.8 ± 117.6	Variable

### 2.7 Clinical correlations

We examined the relationship between macrolide resistance-associated mutations and clinical or laboratory outcomes (Table 6). Children with MRMP tended to have longer fever duration (median 6.5 vs 4.0 days; Mann–Whitney U, *P* = 0.01; median difference 2.5 days, 95% CI [0.5–4.0]) and longer hospitalization (median 7.0 vs 5.0 days; *P* = 0.03). Persistent cough was more common in MRMP (32.8% vs 0%; Fisher’s exact *P* = 0.04; risk difference 32.8%, 95% CI [12.5–52.1]). Inflammatory markers also showed higher medians in MRMP: hs-CRP (11.2 vs 4.8 mg/L; *P* = 0.02; median difference 6.4 mg/L, 95% CI [1.2–11.6]) and LDH (258.5 vs 210.3 U/L; *P* = 0.01; median difference 48.2 U/L, 95% CI [15.0–81.5]). WBC and platelet counts trended higher, but effect estimates had wide CIs due to the very small MSMP sample. Importantly, treatment failure or switch was observed in 20.9% of MRMP but not in MSMP (risk difference 20.9%, 95% CI [5.9–35.9], Fisher’s exact *P* = 0.02).

**Table 6:** Clinical and diagnostic correlations of macrolide resistance.

Clinical/Laboratory marker	MRMP (*n* = 67)	MSMP (*n* = 4)	Effect size (95% CI)	*P* value
Duration of fever (days)	6.5 (5–8)	4.0 (3–5)	+2.5 days (0.5–4.0)	0.01
Length of hospital stay (days)	7.0 (6–9)	5.0 (4–6)	+2.0 days (0.4–3.5)	0.03
Persistence of cough, n (%)	22 (32.8%)	0 (0%)	RD 32.8% (12.5–52.1)	0.04
hs-CRP (mg/L)	11.2 (6.0–16.5)	4.8 (3.5–6.1)	+6.4 (1.2–11.6)	0.02
LDH (U/L)	258.5 (230.0–298.0)	210.3 (180.0–225.5)	+48.2 (15.0–81.5)	0.01
WBC (×10^9/L)	7.8 (6.5–9.0)	6.4 (5.8–7.2)	+1.4 (–0.2–3.0)	0.05
Platelets (×10^9/L)	275.5 (230.0–315.0)	225.0 (210.0–240.0)	+50.5 (10.0–91.0)	0.04
Radiographic resolution delayed	19 (28.4%)	0 (0%)	RD 28.4% (9.8–47.0)	0.03
Treatment failure/switch, n (%)	14 (20.9%)	0 (0%)	RD 20.9% (5.9–35.9)	0.02

RD, Risk Difference; CI, Confidence Interval.

## 4 Discussion

*M. pneumoniae* plays a major role in CAP worldwide and is among the leading contributors in China. Examining the demographic and clinical characteristics of pediatric populations is essential, as these factors influence both the presentation of *M. pneumoniae* pneumonia and the interpretation of antimicrobial resistance trends (26). In the present study, most patients aged 3–12 years presented with fever and cough. The median hospitalization duration (7 days) and frequent radiographic involvement reflect the significant disease burden, and the sex distribution was nearly balanced. These findings are broadly consistent to other pediatric and adult cohorts in the region, although the prevalence of resistance and clinical burden varies markedly by location (27, 28). For instance, a study from Jeju Island, Korea, analyzing 107 pediatric patients, reported a relatively low prevalence of macrolide-resistant *MP* (10.3%), with A2063G as the only resistance mutation detected (29). Despite the lower resistance, the demographic and clinical spectrum of affected children, including age and symptomatology, was comparable to our population. By contrast, a large multicenter study in Chinese adults demonstrated a much higher macrolide resistance prevalence, exceeding 70%, and highlighted cough and fever as the most common presenting symptoms across resistant and susceptible groups (1). Tsai et al. (30) emphasized that macrolide resistance in Asia has increased rapidly since 2018, now representing a substantial clinical challenge. The persistence of fever and delayed recovery noted in Taiwan mirrors the extended febrile course observed in our cohort. Nevertheless, the high prevalence of macrolide resistance observed in this study (94.4%) should be interpreted in the context of the study design. The dataset consisted exclusively of hospitalized pediatric CAP cases with confirmed *M. pneumoniae* infection, and did not include information on the total number of CAP cases screened or those testing negative. As a result, the proportion of MP-positive cases among all CAP presentations could not be determined, and selection bias cannot be excluded. Therefore, the reported resistance prevalence reflects a selected hospital-based cohort rather than a population-level estimate.

Despite universal macrolide administration, many patients received macrolides as first-line therapy, with erythromycin and azithromycin being the predominant choices. *β*-lactams have no *in vitro* activity, indicating that these prescriptions likely reflect empirical CAP management rather than pathogen-directed therapy. Adjunctive antibiotics were administered in nearly 60% of cases, while corticosteroids were prescribed in over half of the patients, particularly in those with persistent fever or severe radiographic involvement. These patterns reflect both the challenges of managing MRMP and the variability in treatment approaches reported across Asia. Han et al. (31) highlighted the role of corticosteroids as adjunctive therapy in severe or refractory MP in Korea, noting improved clinical outcomes when combined with antibiotics. Yang et al. (32) further emphasized that early corticosteroid therapy in pediatric MP was associated with faster resolution of fever and respiratory symptoms, supporting our observation that immunomodulation was frequently employed in patients with more severe clinical courses. In contrast, Sun et al. (3) found that adjunctive antibiotic use in Chinese children was more conservative, with therapy modifications occurring primarily in the setting of confirmed resistance or poor clinical response. Compared with their findings, the relatively high frequency of adjunctive antibiotic use in our cohort suggests a more aggressive treatment strategy, possibly reflecting local clinical practice patterns, higher clinical severity, or concern for bacterial co-infection (7, 33–35).

The MIC profile of the study cohort aligns with recent Chinese pediatric cases showing very high macrolide MICs and widespread resistance. Jia et al. (36) reported 100% macrolide resistance in 62 pediatric isolates with rising azithromycin MICs over time, and uniformly low MICs for tetracyclines/fluoroquinolones in Beijing from 2021 to 2023, closely mirroring our high ERY/AZM MIC50–90 values (ERY MIC50/90 126/512 μg/mL; AZM 16/126 μg/mL). These findings are also consistent with multicenter Chinese study that mapped high macrolide MICs across several regions from 2017 to 2018, and linked them to classic 23S rRNA mutations (37). However, Wang et al. (11) reported susceptibility datasets with mixed patterns in Japan. According to the authors, during 2017–2020, MRMP isolates typically had ERY MICs 16 to >128 μg/mL and AZM 64 to >128 μg/mL, while all isolates remained susceptible to minocycline/levofloxacin, but in some series slightly lower than, our ERY/AZM MIC90s. As reported in the U.S. by Leber et al. (38), resistance was far less common, where contemporary surveillance reported MRMP rates around 2%–10% with correspondingly low macrolide MICs in the susceptible majority, contrasting to our high MIC50/90 pattern. Nevertheless, the magnitude of the macrolide MICs in our study is coherent with the dominance of 23S rRNA domain V mutations (especially A2063G/A2064G), which confer high-level macrolide resistance and drive the MIC shifts seen in Asia (39).

The mutation profile of our study cohort aligns with the East-Asian pattern of A2063G predominance. For example, a large pediatric series from China found that 92.4% of isolates carried domain V mutations, overwhelmingly A2063G, closely mirroring the overall resistance burden and dominant genotype reported in our study cohort (13). Moreover, during the 2023 MP resurgence in Beijing, clinical and molecular surveillance showed very high MRMP rates with A2063G as the signature mutation (40), reinforcing that epidemic waves in China are accompanied by expansion of A2063G-harboring lineages, consistent with the 81.7% A2063G rate in our study. Korea’s recent nationwide upsurge reported a rising frequency of A2063G among pediatric cases in 2014–2024, echoing the dominance of this allele in our study while highlighting regional synchronicity in genotype shifts (41). In contrast, contemporary U.S. surveillance in Ohio, from 2023 to 2024 detected only 2.4% MRMP among nearly 1,000 positives (38), illustrating a stark geographic gradient; the 94.4% mutation rate and A2063G predominance in our study are far higher than U.S. baselines but align with East-Asian epidemiology.

In addition, a recent meta-analysis by Darazam et al. (42) confirms this gradient: pooled A2063G prevalence ∼67% globally, ∼77% in Asia versus ∼10% in the Americas—placing our A2063G (81.7%) slightly above the Asian average but well within expected regional ranges. Besides, subsequent studies establish that A2063G/A2064G in 23S rRNA domain V produces high-level macrolide resistance and large MIC shifts (43), directly supporting the phenotype–genotype concordance observed in the present study. Because most isolates harbored A2063G/A2064G mutations, the downstream laboratory (CRP/LDH) and clinical (fever, LOS, treatment failure) signals observed in our study are consistent with genotype-driven macrolide non-response described in recent pediatric studies (44). Although the A2063G mutation was associated with higher MICs and poorer clinical response, the current data cannot establish a direct causal link between mutation type, resistance phenotype, and clinical deterioration. Additional functional and longitudinal studies are required to confirm this relationship and rule out confounding host or treatment factors.

Laboratory markers in pediatric *MP* mainly reflect host inflammation and tissue injury rather than direct bacterial load (45). In this study, the pattern of mildly elevated hs-CRP and LDH with otherwise unremarkable liver indices in the study mirrors reports that systemic inflammation in pediatric MP is common, but that higher CRP/LDH thresholds tend to mark severe or refractory disease. In a diagnostic meta-analysis study of LDH for refractory MP, cut-offs near 379–530 U/L best discriminated refractory cases, well above our cohort median, supporting that our overall biochemical profile reflects non-refractory illness in most children (46). Consistently, a prospective cohort study found LDH >379 U/L and D-dimer >0.64 mg/L were optimal predictors of severe MP; again, our LDH center fell below these risk thresholds (47). A 2024 analysis linked LDH >393 U/L to necrotizing pneumonia and pulmonary consolidation risk, reinforcing LDH as a lung-injury marker; our values sit in the mild-elevation range by that standard (48). Additionally, in children with CAP, procalcitonin is typically low in *M. pneumoniae* compared with pyogenic bacterial CAP, aligning with our sparse PCT testing and the expectation of low PCT in MP (49). Wang et al. (46) underpins the interpretation that the modest LDH/CRP elevations in the present study reflect inflammation without widespread refractory disease.

The MRMP cases had longer fever, longer hospitalization, higher hs-CRP and LDH, more persistent cough, delayed radiographic resolution, and more treatment failure/switches. This pattern closely matches a meta-analysis study which reported that MRMP is associated with 1.7 days longer fever and 1.6 days longer hospital stay, confirming the direction and magnitude of our observed effects (12). Studies from epidemic settings also report more frequent therapy changes and prolonged fever/hospitalization in MRMP (38), consistent with the higher switch rate in the present study. A recent pediatric work on macrolide-unresponsive MP shows longer fever/hospital stays and higher inflammatory markers such as IL-6, D-dimer, and LDH/albumin ratios and slower radiographic resolution (50), aligning with the increased hs-CRP/LDH and delayed clearance on imaging found in our study. Furthermore, in a large contemporary cohort, fever duration and hospitalization varied by treatment path in settings with high MR prevalence (89%), and escalation beyond macrolides was common, supporting our observation of treatment failure/switch in MRMP (16). The MRMP analysis reported by Chen et al. (12), anchors the correlation between macrolide resistance and worse short-term clinical outcomes, which the present study replicates. Importantly, the observed differences between MRMP and MSMP cases cannot be attributed solely to macrolide resistance. Clinical outcomes in *M. pneumoniae* infection are influenced by multiple factors, including baseline disease severity, host immune response, timing of presentation, corticosteroid use, adjunctive antibiotic therapy, and clinician decision-making (51, 52). Saraya (53) emphasizes that disease manifestations are not solely driven by bacterial burden or resistance status, but are strongly influenced by host–pathogen interactions and epidemiological context. This supports our interpretation that observed differences between MRMP and MSMP cases may not be directly attributable to macrolide resistance alone, and reinforces the potential for confounding and misclassification in clinical outcome assessment. Moreso, given the absence of multivariable adjustment and the extremely small comparator group, these findings should be interpreted as descriptive, non-causal observations rather than evidence of an independent effect of resistance.

Nevertheless, the definition of treatment failure in the present study may be inherently nonspecific. In *M. pneumoniae* infection, prolonged fever and persistent respiratory symptoms are often driven by host immune response and inflammatory processes rather than direct bacterial burden (54). Therefore, clinical non-response to macrolide therapy may reflect the natural disease course, immune-mediated pathology, or co-infection, rather than antimicrobial resistance alone. This introduces the possibility of misclassification. Consequently, treatment failure should not be interpreted as a direct proxy for macrolide resistance in this cohort. Besides, as this was a hospital-based cohort including only admitted patients, the findings may not be generalizable to milder or outpatient pediatric populations. Also, no causal inference can be drawn from these observations.

### 4.1 Limitations of the study

This study has several limitations that should be acknowledged. First, the extremely small MSMP comparator group (*n* = 4) represents a major limitation. This imbalance reduces statistical power and increases the likelihood of both type I and type II errors, leading to unstable estimates. As a result, statistical comparisons should be interpreted cautiously and primarily as descriptive observations. For instance, we used exact tests and reported effect sizes with CI to minimize bias, but the results should still be interpreted with caution. Nevertheless, observed associations should be interpreted as indicative trends rather than definitive causal relationships. Secondly, this was a single-center retrospective study, and the findings may not fully reflect the epidemiology of MP infections in other regions of China. Thirdly, antimicrobial susceptibility testing was restricted to macrolides and β-lactams, and did not include tetracyclines or fluoroquinolones, which remain relevant alternatives in older children and adults. Fourthly, although we sequenced the 23S rRNA domain V to identify key resistance mutations, ribosomal proteins L4 and L22 implicated in macrolide resistance were not sequenced, which limits mechanistic resolution and may overlook non-23S contributions to resistance and clinical phenotype. Furthermore, we did not perform long-term follow-up after discharge; therefore, the persistence of symptoms and radiographic findings beyond hospitalization could not be evaluated. Despite these limitations, the study provides valuable insights into the clinical impact of macrolide resistance in pediatric MP-specific pneumonia, linking molecular and laboratory findings with treatment outcomes in a real-world setting. However, no causal inference can be drawn from these observations. In addition, denominator data for all pediatric CAP cases during the study period were not available, as the dataset included only confirmed *M. pneumoniae* cases. This limits the ability to assess the proportion of MP-positive cases and introduces potential selection bias, thereby affecting the generalizability of the findings.

## 5 Conclusions

Macrolide resistance was nearly universal in this pediatric cohort and was driven predominantly by 23S rRNA A2063G mutations, accompanying very high MICs to erythromycin and azithromycin. Although baseline inflammatory markers were only modestly elevated, MRMP was associated with longer fever and hospitalization, higher hs-CRP and LDH, more persistent cough, delayed radiographic resolution, and greater likelihood of treatment failure or switch. These findings link genotype to phenotype and underscore the clinical relevance of resistance beyond the laboratory. In settings where MRMP prevalence is high, empiric macrolide monotherapy may be insufficient for a substantial proportion of children, and early escalation to alternative agents or adjunctive immunomodulation should be considered when the clinical response is delayed. Our single-center design and small MSMP comparator limit precision, but the direction and magnitude of effects were consistent with prior evidence. While MRMP was observed to be associated with prolonged clinical course and higher inflammatory markers, these findings should be interpreted cautiously due to study design limitations, including the small comparator group and lack of multivariable adjustment. Larger, prospective studies are needed to confirm the independent clinical impact of macrolide resistance.

## Data Availability

The original contributions presented in the study are included in the article/Supplementary Material, further inquiries can be directed to the corresponding author/s.
