# Correction: Macrolide-resistant *Mycoplasma pneumoniae* in children: clinical features, treatment response, and outcomes in a retrospective cohort from China

**DOI:** 10.3389/fped.2026.1992590

**Published:** 2026-10-08

**Authors:** Lulu Zheng, Huaqing Liu, Cunxin Xu, Feifei Song, Shenggang Ding

**Affiliations:** 1 Department of Pediatrics, Anhui Zhongke Gengjiu Hospital, Hefei, Anhui Province, China; 2 Department of Pediatrics, First Affiliated Hospital of Anhui Medical University, Hefei, China; 3 Anhui Provincial Children’s Hospital, Hefei, Anhui Province, China

**Keywords:** 23S rRNA mutations, antimicrobial resistance, clinical outcomes, minimal inhibitory concentration, *Mycoplasma pneumoniae*, pediatric pneumonia

In the published article, there was an error in the reported study period. The study period was incorrectly stated as October 2023 to September 2024. The correct study period is July 2023 to July 2024. This was an error in reporting the study dates only. The underlying patient records and data analyzed correspond to the correct study period, and this correction does not affect the sample size, analyses, results, interpretation, or conclusions of the article.

A correction has been made to the Abstract, Methods, and to the section Materials and methods, subsection 2.1 Study design and population, Paragraph 1:

“Methods: A retrospective cohort study was conducted among 71 pediatric patients with confirmed M. pneumoniae CAP between July 2023 and July 2024. Macrolide resistance was assessed using 23S rRNA domain V sequencing and broth microdilution minimum inhibitory concentration (MIC) testing. Clinical features, laboratory markers, treatment response, and outcomes were descriptively compared between macrolide-resistant M. pneumoniae (MRMP) and macrolide-susceptible M. pneumoniae (MSMP) cases.”

“This retrospective single-center clinical study was conducted among pediatric patients diagnosed with CAP who attended Anhui Zhongke Gengjiu Hospital in Anhui, China, between July 2023 and July 2024. Eligible participants were children with clinical symptoms and chest radiographic findings consistent with CAP and a confirmed diagnosis of MP infection by culture and/or polymerase chain reaction (PCR). Patients were consecutively enrolled among hospitalized pediatric CAP cases during the study period.”

The original version of this article has been updated.

